# Impact of Soil Disinfestation on Fungal and Bacterial Communities in Soil With Cucumber Cultivation

**DOI:** 10.3389/fmicb.2021.685111

**Published:** 2021-08-19

**Authors:** Yan Wang, Yujie Jin, Ping Han, Jianjun Hao, Hongyu Pan, Jinliang Liu

**Affiliations:** ^1^College of Plant Science, Jilin University, Changchun, China; ^2^Beijing Research Center for Agricultural Standards and Testing, Beijing Academy of Agriculture and Forestry Science, Beijing, China; ^3^School of Food and Agriculture, The University of Maine, Orono, ME, United States

**Keywords:** high-throughput sequencing, Illumina, enzymatic activities, soil restoration, disinfectants

## Abstract

Soil treatment with disinfectants has been used for controlling soilborne phytopathogens. Besides suppressing specific pathogens, how these disinfectants impact soil health, especially soil microbial communities, is yet to be systemically determined. The objectives of this study were to examine the effects of three representative disinfectants, including the dazomet fumigant, fenaminosulf fungicide, and kasugamycin antibiotic on chemical properties, enzymatic activities, and microbial communities in soil for cucumber cultivation. Results showed that 14 days after soil treatment with these chemicals, residual content of dazomet and kasugamycin quickly declined in soil and were undetectable, while fenaminosulf residues were found at 0.48 ± 0.01 mg/kg. Total nitrogen and total carbon increased in soil after dazomet treatment. Urease and sucrase activities were significantly restrained after disinfectant application. The disinfectants did not significantly change the taxon of predominant bacteria and fungi but altered the relative abundance and diversity of soil microbiome, as well as microbial interspecific relationships. Moreover, cucumber cultivation enhanced the overall soil microbial diversity and enzymatic activities, which diminished the difference of soil microbiome among four treatments. The difference in soil microbial diversity among the four treatments became smaller after planting cucumber. Thus, soil microbial communities were affected by soil disinfectants and gradually recovered by cucumber application.

## Introduction

Soil microbiome is a key contributor to soil properties, which includes various beneficial microorganisms, as well as pathogens (Dangi et al., 2015). Soilborne pathogens have threatened the agricultural industry, which caused dramatic productivity reduction and destructive economic loss. For example, continuous cropping of cucumber can be constrained by Fusarium wilt, which has been reported to be one of the most destructive diseases in cucumber production in China and worldwide (Jaiswal et al., 2014; Zhao et al., 2017). However, many soil microbiomes are beneficial and enhance soil health and plant health, which are responsible for multifaceted functions in soils including cycling carbon (C) and nitrogen (N), degrading pollutants and pesticides residing in soil, and promoting plant defense against pathogens (Kibblewhite et al., 2008; Yim et al., 2017). Such beneficial microorganisms from bacterial genera *Pseudomonas*, *Sphingomonas*, and *Streptomyces* and fungal genera *Trichoderma*, *Chaetomium*, and *Gliocladium* have been reported from previous studies (Raaijmakers et al., 2009; Meng et al., 2012; Nicola et al., 2017; Huang et al., 2020). The goal of maintaining healthy soil is to largely reduce pathogens and sustain or enhance beneficial microorganisms. To examine the integrity of soil microbiome, microbial structure and diversity have been used as indicators (Sharma et al., 2010), which are closely related to soil ecosystem function (Maron et al., 2018).

To control soilborne diseases, soil can be treated with various methods, such as steaming, applying biocontrol agents and chemical pesticides, and soil fumigation (Zhao et al., 2017; Zhou et al., 2017; Ye et al., 2020). Fumigants used frequently include 1,3-dichloropropene (1,3-D), chloropicrin, methyl isothiocyanate (MITC), and the MITC generator dazomet (Van Wambeke, 2007). Fungicides such as carbendazim, azoxystrobin, and thiophanate methyl have been used for soil disinfection to control soilborne diseases (Zhao et al., 2016; William et al., 2021).

Soil microorganisms respond differently to disinfectants used for soil treatments depending on the chemical properties (Nicola et al., 2017; Zhang et al., 2017). This can be judged by the change of composition, abundance, and diversity of microbial communities affected by disinfectants (Zhao et al., 2016; Nicola et al., 2017). Such impacts of disinfectants may affect functional diversity and soil quality with changes in soil physical and chemical properties and enzymatic activities and changes in the abundance of beneficial soil microorganisms (Wu et al., 2014; Peilei et al., 2020).

Soil disinfectants and their degradation metabolites may have some negative effects on soil microbial communities and soil environment, especially for beneficial microbes (Dangi et al., 2015; Zhang et al., 2017). It has been reported that repeated iprodione applications exert negative effects on soil enzymatic activities associated with the cycling of C, N, phosphorus (P), and sulfur (S) and decline relative abundances of function bacteria, such as Chloroflexi and Acidobacteria (Zhang et al., 2017). However, disinfectants reduce the population of many beneficial microbes for a short period; these effects may be positive after all because soil microorganisms have the ability to recover after some disinfectant application (Dianli et al., 2019). Soil fumigation with dazomet reduces apple replant disease by reducing three potentially pathogenic fungi, *Hyonectria* sp., *Pyrenochaeta* sp., and *Mortierella* sp. (Nicola et al., 2017). Relative abundances of some beneficial microorganisms increase 19 months after the soil treatment, such as fungi *Chaetomium* spp. and bacteria *Streptomyces* spp. and *Bacillus* spp., which suppress plant pathogens or promote plant growth (Nicola et al., 2017). Application of 1,3-dichloropropene significantly reduced soil bacterial community diversities, and then soil bacterial community diversities gradually recovered; no significant difference was found compared to the control group (Dianli et al., 2019).

Our best interest is to maintain the soil in good condition after disinfectant application (Kibblewhite et al., 2008). If soil properties were affected, restoring or recovering soil health is the priority by additional inputs and environmental disturbance (Kibblewhite et al., 2008; Fang et al., 2020). Most likely, the negative effects on the microbial community may be transitory and will disappear shortly, as disinfectants are degraded rapidly in soil (Fang et al., 2020). Plant cultivation can also restore soil properties (Bárcenas-Moreno et al., 2011; Fang et al., 2020). However, these need to be determined in the field for different disinfectants.

Although various disinfectants are being used in cucumber cultivation in China, including fenaminosulf (fungicide), kasugamycin (antibiotic), and dazomet (fumigant), how these disinfectants and cucumber planting affect the soil microecology is not clear. The aims of this study were to examine microbiome responses of soil treated with the above disinfectants and the effect of cucumber cultivation on the restoration of soil health.

## Materials and Methods

### Soil Treatment

This study was carried out in a greenhouse of Jilin Vegetable Flower Science Research Institute, Changchun Experimental Station in Jilin, China (125.22°E, 43.49°N) in 2018. Cucumber had been grown for 3 years from 2016 to 2018. The soil is composed of 5% coarse sand, 64% fine sand, 25% silt, and 6% clay. Soil organic matter content is 3.81%. Available N, available P, and available potassium are 51.2, 6.27, and 68.9 mg⋅kg^–1^, respectively. Four soil treatments were applied, including A: non-treated control; B: 70% fenaminosulf SP (Sichuan Runer Technology Co., Ltd.) at 5.2 kg⋅ha^–1^; C: 2% kasugamycin WP (Meibang Pesticide Co., Ltd., Shaanxi, China) at 12.5 kg⋅ha^–1^; and D: 98% dazomet GR (Shunyi Co., Ltd., Taizhou, China) at 400 kg⋅ha^–1^ based on the maximum recommended doses. Soil fumigation with dazomet was conducted on April 12, 2018, followed by irrigating and covering the soil with plastic film. The plastic was removed after 7 days. Completely randomized block design with three replications was applied. Plot size was 4 m × 5 m. Cucumber seedlings were prepared and transplanted into plots 14 days after soil treatment, with 100 seedlings per plot. Plants were harvested 110 days after soil treatment.

### Soil Sampling

Around 500 g of bulk soil was sampled at a depth of 0–20 cm by compositing five subsamples in each plot 55 and 110 days after soil treatment, respectively. The soil samples were transported to the laboratory in an ice-cooled container and then sieved through 2-mm mesh to remove plant debris. Each sample was divided into three parts: part 1 was air-dried for chemical analysis, part 2 was stored at 4°C for analyzing enzymatic activities and disinfectant residues, and part 3 was stored at −80°C for DNA extraction.

### Soil Analyses

Soil pH was determined in 1:2.5 soil/deionized water suspensions using a pH meter (PHS-25, Leici, Shanghai, China). Soil total nitrogen (TN) and soil total carbon (TC) concentrations were measured by an Elemental Analyzer (ECS 4024 CHNSO, Costech Inc., Cernusco sul Naviglio, Italy). Soil urease (UE), alkaline phosphatase (ALP), sucrase (SC), and catalase (CAT) were measured using a UE assay kit, an ALP assay kit, an SC assay kit, and a CAT assay kit, respectively (Nanjing Jiancheng Bioengineering Institute, Nanjing, China) following the manufacturer’s instructions. Each treatment was replicated three times, and the experiment was repeated once. The data were expressed on a dry-weight basis. Geometric mean of enzymatic activities (GMEA) was calculated as GMEA = (UE × ALP × SC × CAT)^1/4^ for enzymatic activities.

### Detection of Disinfectant Residues in Soil

Soil samples were processed for chemical extraction using the QuEChERS methods with slight modification (Abd-Alrahman, 2014). Five grams of soils were crushed and placed in a 50-ml centrifuge tubes, followed by adding different chemicals depending on specific disinfectants. For dazomet extraction, 10 ml of mixture of acetonitrile and water (9:1, v/v) was added in the tube. For fenaminosulf extraction, a mixture of acetonitrile and water (8:2, v/v) was used, and for kasugamycin extraction, a mixture of acetonitrile and water (7:3, v/v) was used. The centrifuge tube was shaken on an oscillator at 220 rpm and 25°C for 20 min and treated by ultrasound for 10 min, followed by centrifugation for 5 min at 7,168 *g* and 25°C. The supernatant was passed through a 0.22-μm filter membrane and transferred into an autosampler vial for high-performance liquid chromatography (HPLC) analysis.

For detecting disinfectants, a liquid chromatography system (1260 series, Agilent Technologies, Santa Clara, CA, United States) equipped with an autosampler, a binary pump, and a diode array detector (DAD-UV–visible) was used. Compound separation was carried out on a carbon 18 (250 mm × 4.6 mm, 5 μm) column. For dazomet detection, the mobile phase was acetonitrile:water (10/90, v/v) at a flow rate of 1.0 ml/min, and 10-μl injection volume was chosen; detection wavelength was set at 254 nm, and the retention time was about 5.0 min. For fenaminosulf detection, the mobile phase was acetonitrile:water (70/30, v/v) at a flow rate of 1.0 ml/min, and 20-μl injection volume was chosen; detection wavelength was set at 391 nm, and the retention time was about 4.0 min. For kasugamycin detection, the mobile phase was 1% sodium dodecyl-benzenesulfonate (SDS) aqueous solution:acetonitrile (4/1, v/v) at a flow rate of 1.0 ml/min, and 5-μl injection volume was chosen; detection wavelength was set at 251 nm, and the retention time was about 15.08 min. Data acquisition and processing were performed using the Chemstation software (Agilent Technologies, Rev. B.04.03-SP1).

To validate and evaluate the accuracy of the extraction method, the recovery rate and detection limit of the three disinfectants in soils were determined (Supplementary Table 1). The average recovery rate ranged from 69.4 to 95.5% in soil, and the relative standard deviation (RSD) in soil was less than 11%. These results indicated that the approach was suitable for analyzing disinfectant residues in soil.

### High-Throughput Sequencing and Bioinformatic Analysis

Total genomic DNA was extracted from soil samples using the EZNA^®^ stool DNA Kit (Omega Bio-tek, Norcross, GA, United States) according to the manufacturer’s instructions. DNA quality was determined using a NanoDrop 1000 spectrophotometer (Thermo Scientific, Hudson, NH, United States) and on 0.8% agarose gels. The V3-V4 hypervariable region of 16S rDNA for bacteria was amplified using the forward primer 338F (5′-ACTCCTACGGGAGGCAGCAG-3′) and the reverse primer 806R (5′-GGACTACHVGGGTWTCTAAT-3′), and the internal transcribed spacer (ITS) 1 region for fungi was amplified using the forward primer ITS1-F (5′-CTTGGTCATTTAGAGGAAGTAA-3′) and the reverse primer ITS2 (5′-TGCGTTCTTCATCGATGC-3′) by PCR according to Li et al. (2015). PCR productions were subjected to high-throughput sequencing by Beijing Allwegene Tech, Ltd. (Beijing, China), using the Illumina MiSeq PE300 sequencing platform (Illumina, Inc., CA, United States).

All sequences were first conducted with the QIIME package (Quantitative Insights Into Microbial Ecology) (v1.2.1) for Illumina sequencing data through quality filtering and chimera removal, and the retained effective tags were used to perform operational taxonomic unit (OTU) and species annotation. The unique sequence was classified into OTUs under the threshold of 97% identity using UCLUST. Chimeric sequences were identified and removed using Usearch (version 8.0.1623). The taxonomy of each 16S rRNA and ITS gene sequences was analyzed by UCLUST against the Silva119 16S rRNA and UNITE database using a confidence threshold of 90%. The raw readings for bacteria and fungi were jointly deposited into the NCBI Sequence Read Archive (SRA) database under BioProject accession number PRJNA392356. Microbial community structure was analyzed using permutational multivariate analysis of variance (PERMANOVA) based on the Bray–Curtis distance metrics with 999 permutations using the R statistical software (version 3.6.0). For multivariate analysis of microbial communities, non-metric multidimensional scaling (NMDS) and Redundancy analysis (RDA) were conducted using CANOCO, version 5. To demonstrate the relationship of different genera among four treatments for different sampling times, the co-occurrence network was performed using the top 20 abundant genera of bacterial and fungal communities based on Spearman’s rank analysis (*ρ* > 0.6) and significant correlations (*p* < 0.05) and was visualized with the Gephi (Li and Wu, 2018).

### Statistical Analysis

Statistical analysis was done by using SPSS Version 24.0 (IBM SPSS Statistics, Armonk, NY, United States). One-way repeated measurement was employed for differences across all sampling times at a significance level α = 0.05. One-way analysis of variance was employed to compare treatment effects. Means were separated by Fisher’s LSD tests at a significance level α = 0.05.

## Results

### Chemical Properties of Soils

Total carbon, total nitrogen, and pH of soils were determined under the treatment of soil disinfectants (Table 1). In the non-treated soil, TC and TN increased after cucumber planting (*p* < 0.05), but C/N ratio did not change (*p* > 0.05). The change of TC and TN was not significant under three disinfectant applications (*p* > 0.05). Compared to the non-treated soil, TC, TN, C/N, and pH values slightly decreased by fenaminosulf treatment 55 days after soil treatment but went up 110 days after soil treatments (*p* < 0.05). Treatments with kasugamycin and dazomet had opposite results except pH, increased 55 days after soil treatment and decreased 110 days after soil treatments (*p* < 0.05). TC and TN increased after planting in dazomet-treated plots, and TC was slightly increased by kasugamycin. Regardless of TN and TC changes, C/N ratio was not affected by any of the treatments (*p* > 0.05). pH values did not significantly change during the season in any of the treatments (*p* > 0.05; Table 1).

**TABLE 1:** Chemical properties of soils treated with disinfectants, 2018.

	Sampling time	Non-treated	Fenaminosulf	Kasugamycin	Dazomet
TC (g/kg)	April 26	57.1 ± 13.3 a	57.8 ± 16.5	47.9 ± 11.7	61.0 ± 7.1
	June 6	34.4 ± 6.5 bC	40.6 ± 5.1 C	50.9 ± 9.8 B	68.6 ± 4.7 A
	July 31	37.4 ± 2.2 bB	51.5 ± 14.0 AB	41.1 ± 10.2 B	67.5 ± 7.0 A
TN (g/kg)	April 26	4.3 ± 0.8 a	4.3 ± 1.0	3.6 ± 0.9	4.4 ± 0.2
	June 6	2.7 ± 0.3 bB	3.1 ± 0.3 B	3.6 ± 0.7 B	4.8 ± 0.2 A
	July 31	2.9 ± 0.1 bB	3.8 ± 0.9 AB	3.0 ± 0.6 B	4.9 ± 0.3 A
C/N	April 26	13.6 ± 0.7	13.3 ± 0.8	13.4 ± 0.8	13.8 ± 1.4
	June 6	12.6 ± 1.0	13.0 ± 0.4	14.0 ± 0.7	14.2 ± 1.1
	July 31	13.0 ± 0.2	13.6 ± 1.0	13.4 ± 0.9	13.9 ± 0.5
pH	April 26	6.9 ± 0.1 A	6.8 ± 0.1 A	6.6 ± 0.1 B	6.5 ± 0.0 B
	June 6	6.6 ± 0.4	6.7 ± 0.2	6.3 ± 0.3	6.4 ± 0.4
	July 31	6.8 ± 0.0	6.7 ± 0.1	6.4 ± 0.3	6.7 ± 0.3

*Values represent mean ± standard deviation of triplicate measurements. Means were separated by comparing treatments on each sampling date (marked with lowercase letters) or comparing data points of sampling times for each measurement within a treatment (marked with capitalized letters). Different letters following the mean values indicate significant differences (*p* < 0.05). No letter means no significant difference. TN, total nitrogen; TC, total carbon; C/N, the ratio of total carbon to total nitrogen.*

*Cucumber was planted on April 26.*

### Soil Enzymatic Activities

**FIGURE 1:**
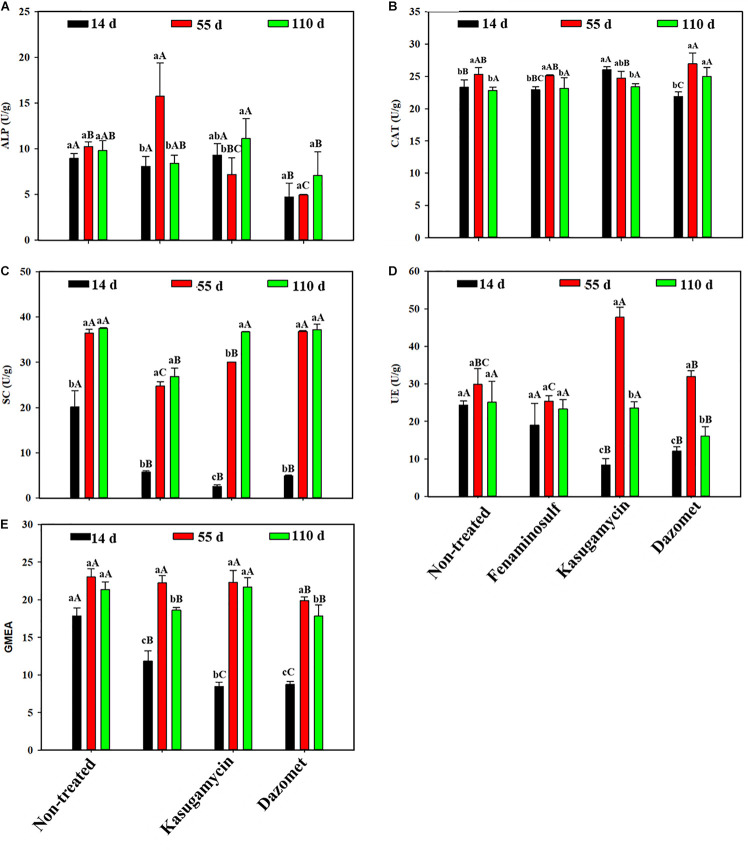
Enzymatic activities in disinfectant-treated soils that were measured by **(A)** alkaline phosphatase (ALP), **(B)** catalase (CAT), **(C)** sucrase (SC), **(D)** urease (UE), and **(E)** geometric mean for the enzyme activities (GMEA). Values represent mean ± standard deviation of triplicates. GMEA = (UE × ALP × SC × CAT)^1/4^. Different lowercase letters indicate significant differences (*p* < 0.05) at different sampling times within a treatment, and different capitalized letters indicate significant differences (*p* < 0.05) among treatments at a particular sampling time.

Soil enzymatic activities varied under different disinfectant treatments and sampling times (Figure 1). After 14 days of soil treatments, activities of SC, UE, and GMEA significantly decreased in all treatments compared to the non-treated control (*p* < 0.05). In addition, activities of ALP and CAT decreased under dazomet treatment, and kasugamycin increased CAT activities. Most enzymatic actives increased with planting cucumber 55 days after soil treatment. Especially SC and GMEA activities showed obvious rising trends in all the treatments. Activities of ALP increased and activities of SC and UE reduced in fenaminosulf. UE activities were promoted but SC activities decreased in kasugamycin. ALP activities and GMEA reduced in dazomet compared to those in non-treated control. Most enzymatic activities showed no difference with those of the controls 110 days after soil treatments (*p* > 0.05). However, SC and GMEA activities in fenaminosulf and UE and GMEA activities in dazomet were lower than those in the controls. Most enzymatic activities reduced by soil treatments bounced back more or less to the level of non-treated control after cucumber cultivation.

### Disinfectant Residues in Soils

Kasugamycin and dazomet concentrations in soil were lower than detection limits of 0.085 and 0.021 mg/kg, respectively. The concentrations of fenaminosulf decreased progressively from 0.48 ± 0.01 to 0.28 ± 0.08 mg/kg.

### Alpha Diversities of Bacteria and Fungi

**FIGURE 2:**
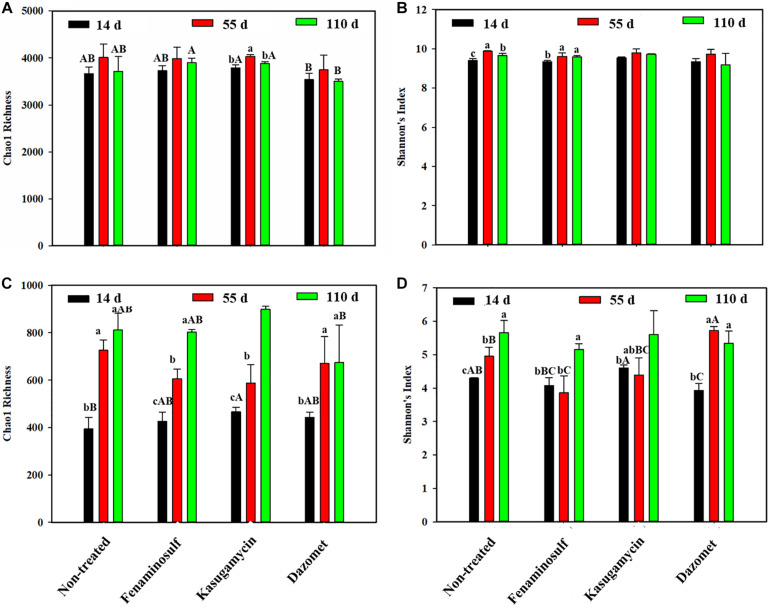
Chao1 richness and Shannon diversity of bacteria **(A,B)** and fungi **(C,D)** in **(A)** non-treated soil and soils treated with **(B)** fenaminosulf, **(C)** kasugamycin, and **(D)** dazomet. Values represent mean ± standard deviation of triplicate measurements. Different lowercase letters in the same column indicate significant differences (*p* < 0.05) at different sampling times in one treatment, and different capital letters indicate significant differences (*p* < 0.05) among different treatments at a particular sampling time.

The change of soil microorganisms was shown under cucumber cultivation and disinfectant application (Figure 2). For bacteria diversities, the Chao 1 richness had no significant difference under any of the disinfectant applications (Figure 2A) (*p* > 0.05). Dazomet reduced soil bacterial abundance. In the presence of cucumber, bacterial diversity, determined by Shannon’s index, went up in the non-treated control and fenaminosulf-treated soils, and Chao 1 richness was increased by kasugamycin. However, no significant difference was found in Chao 1 richness and Shannon indexes between treatments with disinfectants and non-treatment control after planting cucumbers (*p* > 0.05).

For fungal diversity, changes of Chao 1 richness and Shannon diversity index were influenced by disinfectant application and planting cucumbers (Figures 2C,D). Chao1 richness and Shannon index were significantly higher in kasugamycin than those in other treatments (*p* < 0.05). Shannon diversity was found decreased in the dazomet application group. Both Chao1 richness and Shannon index significantly increased after planting cucumbers except the Shannon index of treatments with fenaminosulf and kasugamycin. No significant difference was found on the diversity between the non-treated control and any disinfectant treatment 110 days after soil treatment (*p* < 0.05).

### Community Structures of Bacteria in Response to Disinfectant Application

**FIGURE 3:**
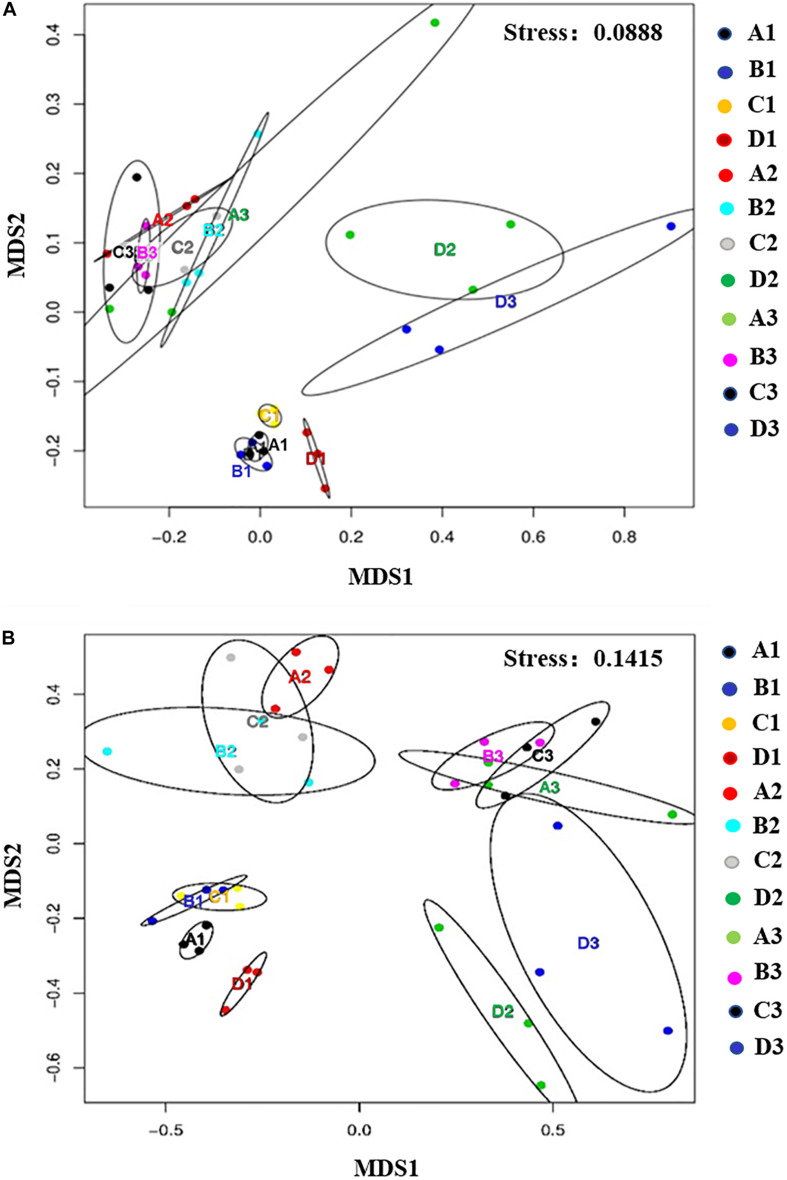
Non-metric multidimensional scaling (NMDS) analysis showed the relationship between the samples in bacterial **(A)** and fungal **(B)** communities. First two dimensions of NMDS ordinations derived from the Bray–Curtis dissimilarity matrices were based on the relative abundance of bacterial and fungal families. A, non-treated; B, fenaminosulf; C, kasugamycin; D, dazomet. Numbers 1, 2, and 3 represent samples collected at 14, 55, and 110 days after soil treatment, respectively. The ellipse in each figure represents a confidence region, which was a multidimensional generalization of a confidence interval. The absence of intersection between ellipses indicates differences among treatments (*p* < 0.05).

Non-metric multidimensional scaling was conducted to examine soil community profiles of bacteria and fungi under disinfectant application and cucumber cultivation (Figure 3). Based on PERMANOVA, after disinfectant application, bacterial communities in dazomet and kasugamycin treatments were found significantly different compared to the non-treated control (*p* < 0.05) but not in fenaminosulf treatment (Table 2). Specific bacterial communities were formed at different sampling times, which were also affected by the type of disinfectant, except that non-treated control and fenaminosulf had closely related bacterial communities (Figure 3). The distances at 110 days after soil treatment shrank after cucumber planting (Pseudo-F = 2.3821, *p* = 0.025).

**TABLE 2:** Permutational multivariate analysis of variance (PERMANOVA) of microbial community structure in soil 14 days after disinfectant treatment.

		Comparison of microbial			
		community structure			
Microorganism	Statistic	A and B	A and C	A and D	A, B, C, and D
Fungi	Pseudo-F	4.9005	3.1247	7.6195	2.7193
	*p*-value	0.0040	0.0080	0.0030	0.0020
Bacteria	Pseudo-F	3.5234	3.6853	2.5245	2.1055
	*p*-value	0.1570	0.0030	0.0240	0.0010

*Non-treated (A), fenaminosulf (B), kasugamycin (C), and dazomet (D).*

**FIGURE 4:**
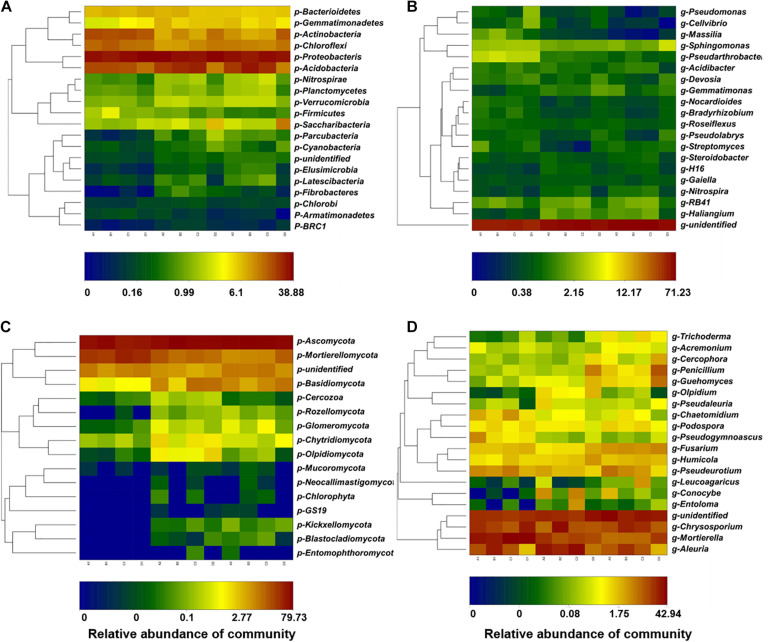
Taxonomic profiles of the bacterial and fungal communities at the phylum and genus levels. The relative abundances of the top 20 bacterial phyla **(A)** and the top 20 bacterial genera **(B)**. The relative abundances of the top 20 fungal phyla **(C)** and the top 20 fungal genera **(D)**. A, non-treated control; B, soils treated with fenaminosulf; C, soils treated with kasugamycin; D, soils treated with dazomet. Numbers 1, 2, and 3 represent samples collected at 14, 55, and 110 days after soil treatment, respectively.

In the bacterial community, Proteobacteria, Acidobacteria, Chloroflexi, and Actinobacteria were the most abundant phyla, which accounted for 71.5–84.7% of total bacterial communities (Supplementary Table 2). The relative abundance of Firmicutes was increased by fenaminosulf, Gemmatimonadetes were increased by kasugamycin, and Gemmatimonadetes and Acidobacteria were increased and Nitrospirae were significantly decreased by dazomet (Figure 4A) (*p* < 0.05). After cucumber planting, a considerable decline of Actinobacteria and Chloroflexi occurred, whereas distinct increases were observed in Gemmatimonadetes, Nitrospirae, Parcubacteria, Cyanobacteria, Elusimicrobia, and Fibrobacteres for all treatments.

Further analysis was performed by analyzing relative abundance at the genus level. Bacterial genera, especially some potential functional microorganisms, varied depending on the type of disinfectant used (Figure 4B). *Massilia* have functions such as P solubilization, degradation of phenanthrene, and resistance to heavy metals, so they can be potentially beneficial organisms (Yang et al., 2019). *Gemmatimonas*, *Streptomyces*, and *Sphingomonas* are closely related to P metabolism, and *Nitrospira* are closely related to N metabolism (Xun et al., 2021). *Pseudomonas* and *Streptomyces* include potential plant pathogens and antagonists beneficial to plants (Koki and Makoto, 1996; Nicola et al., 2017; Tanya et al., 2020). In fenaminosulf-treated soils, *Massilia* increased, whereas other genera, such as *Gemmatimonas*, *Streptomyces*, and *Nitrospira* decreased. In kasugamycin-treated soils, the predominant groups *Pseudomonas* and *Pseudolabrys* and many other bacteria decreased, with few exceptions, such as *Gemmatimonas* that increased. Dazomet significantly promoted *Pseudomonas* and *Gemmatimonas* and showed a deleterious effect on *Massilia*, *Nitrospira*, and many other bacteria. In the presence of cucumbers, *Pseudomonas*, *Massilia*, *Sphingomonas*, and *Streptomyces* increased, most of which are potentially beneficial bacteria, while *Haliangium* and *Gemmatimonas* decreased.

### Community Structures of Fungi in Response to Disinfectant Application

Fungal communities were examined in soils under different disinfectant treatments. According to PERMANOVA, fungal communities of all treatments were significantly different compared to the non-treated control (*p* < 0.5; Table 2). Specific fungal communities were formed at each sampling time, which were also affected by the type of disinfectant, except that kasugamycin and dazomet treatments resulted in similar fungal communities (Figure 3). The distances among samples at 110 days after soil treatment shrank after cucumber planting (Pseudo-F = 1.6496, *p* = 0.040).

Main fungal phyla were Ascomycota and Mortierellomycota in all soil samples (Figure 4C). Ascomycota was a predominant phylum across all treatments between 47.5 and 79.7%, whose relative abundance was reduced by kasugamycin and dazomet treatments but restored after cucumber cultivation. Conversely, relative abundance of Mortierellomycota was increased by kasugamycin and dazomet treatments and declined in the recovery of cucumber cultivation. Interestingly, fungal phyla such as GS19, Neocallimastigomycota, Entomophthoromycota, Blastocladiomycota, and Kickxellomycota that were under detection level before cucumber cultivation were observed in the samples after planting cucumbers.

Further analysis was performed by analyzing relative abundance at the genus level. The majority genera included *Aleuria*, *Chrysosporium*, and *Mortierella* under all treatments (Figure 4D). Many potential beneficial antagonists are from *Acremonium*, *Penicillium*, *Guehomyces*, *Trichoderma*, and *Mortierella* (Jinu and Park, 2014; Hong et al., 2020; Huang et al., 2020; Li et al., 2020). *Fusarium* spp. can be either phytopathogens or beneficial organisms (Liu and Zhang, 2021). These genera showed significant changes under disinfectant application and cucumber cultivation. Compared with the non-treated control, *Acremonium* decreased and *Guehomyces* increased under fenaminosulf treatment. *Acremonium* and *Pseudogymnoascus* (saprophytes with potential mycorrhizal association) decreased (Heikki et al., 2020) and *Olpidium* (potential plant pathogen) (Filiz and Filiz, 2021) and *Guehomyces* increased in kasugamycin-treated soils. *Aleuria* decreased but *Trichoderma* and *Guehomyces* increased in dazomet-treated soils. Cucumber cultivation increased *Trichoderma*, *Guehomyces*, and *Fusarium* but decreased *Pseudogymnoascus* and *Mortierella*. Fungal pathogens were reduced by fenaminosulf and dazomet, indicating that growth of some plant pathogens was inhibited (Supplementary Figure 1). The beneficial organism *Trichoderma* increased under dazomet treatment or cucumber cultivation, indicating a way of disease suppression. The incidence of fungal disease was 2.3, 0.0, 1.0, and 0.0% under the treatments of non-treated, fenaminosulf, kasugamycin, and dazomet, which confirmed that fenaminosulf and dazomet were effective in reducing or eliminating fungal pathogens.

### Relationships Between Soil Chemical Properties and Microbial Diversities

**FIGURE 5:**
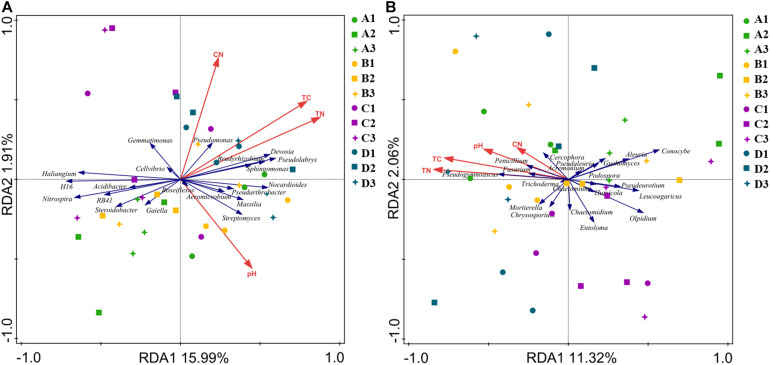
Redundancy analysis biplots of soil bacterial **(A)** and fungal **(B)** community composition. Soil properties included soil total carbon (TC), total nitrogen (TN), soil pH, and carbon/nitrogen ratio (C/N). A, non-treated; B, fenaminosulf; C, kasugamycin; D, dazomet. Numbers 1, 2, and 3 represent samples collected at 14, 55, and 110 days after soil treatment, respectively.

Redundancy analysis biplots were used to determine the relationship between chemical properties and relative abundances of bacterial and fungal communities. The combination of variables explained bacterial (17.9%) and fungal (13.4%) community structure variances (Figure 5). For the bacterial community, the increased relative abundance of *Pseudomonas* was associated with increased TC and C/N, whereas the relative abundance of *Devosia*, *Pseudolabrys*, *Sphingomonas*, and *Bradyrhizobium* was associated with increased TN, but *Nitrospira* was on the opposite way. *Steroidobacter* was associated with decreased TC. *Aeromicrobium* and *Streptomyces* were positively correlated, but *Gemmatimonas* and *Cellvibrio* were negatively correlated with pH (Supplementary Table 3). For the fungal community, the relative abundance of *Pseudogymnoascus*, *Fusarium*, *Penicillium*, and *Cercophora* was positively correlated with TN, TC, pH, and C/N (Supplementary Table 3). In contrast, the relative abundance of *Pseudeurotium* and *Leucoagaricus* was negatively correlated with TC; *Olpidium* was negatively correlated with pH (Figure 5). Disinfectant applications caused changes of relative abundances of functional microorganisms, which were indicated by the chemical properties of the soil because the chemical properties of the soil were closely related to the changes of microorganisms.

### Co-occurrence Network Analysis

**FIGURE 6:**
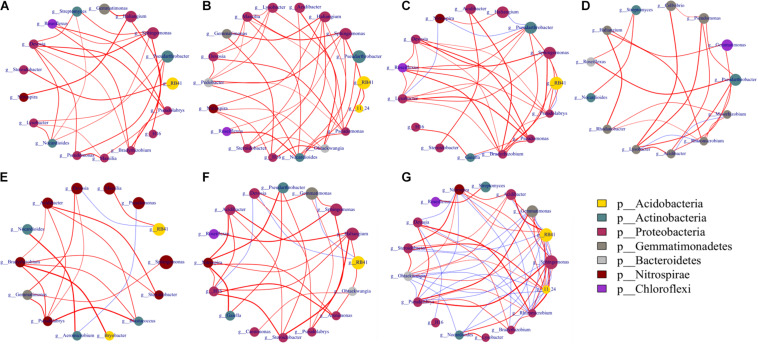
Networks of co-occurring bacterial genera based on correlation analysis. Co-occurrence network was performed using the top 20 abundant genera of bacterial and fungal communities based on Spearman’s rank analysis (*ρ* > 0.6) and significant correlations (*p* < 0.05). Circles represent nodes whose size indicates the relative abundance, the thickness of each edge is proportional to the value of Spearman’s correlation coefficients, and node color represents taxonomy at the phyla level. Edges indicate co-occurrence between nodes colored either blue for negative or red for positive. **(A)** A1, A2, A3; **(B)** B1, B2, B3; **(C)** C1, C2, C3; **(D)** D1, D2, D3; **(E)** A1, B1, C1, D1; **(F)** A2, B2, C2, D2; **(G)** A3, B3, C3, D3. Treatments included A, non-treated control; B, fenaminosulf; C, kasugamycin; D, dazomet. Numbers 1, 2, and 3 represent samples collected at 14, 55, and 110 days after soil treatment, respectively.

**FIGURE 7:**
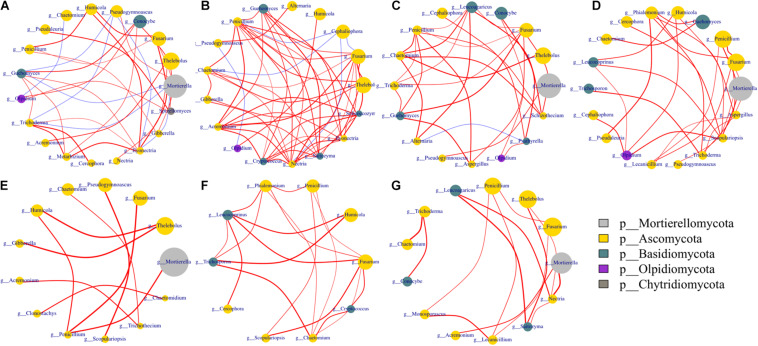
Networks of co-occurring fungal genera. Co-occurrence network was performed using the top 20 abundant genera of bacterial and fungal communities based on Spearman’s rank analysis (*ρ* > 0.6) and significant correlations (*p* < 0.05). The size of circles at nodes represents relative abundance, the thickness of each edge is proportional to the value of Spearman’s correlation coefficients, and node color represents taxonomy at the phyla level. Edges indicate co-occurrence between nodes colored either blue for negative or red for positive. Treatments included A, non-treated control; B, fenaminosulf; C, kasugamycin; D, dazomet. Numbers 1, 2, and 3 represent samples collected at 14, 55, and 110 days after soil treatment, respectively. **(A)** A1, A2, A3; **(B)** B1, B2, B3; **(C)** C1, C2, C3; **(D)** D1, D2, D3; **(E)** A1, B1, C1, D1; **(F)**: A2, B2, C2, D2; **(G)** A3, B3, C3, D3.

Network analysis was performed to explore the interrelationships of microbial taxa in the complex of microbial communities. Soil microbial relationships changed under different disinfectant applications at each sampling or different sampling periods within a soil treatment (Figures 6, 7; Supplementary Table 4) (*ρ* > 0.6, *p* < 0.05). Bacteria had more complex networks than fungi, indicating that soil bacterial taxa had more connections and closer relationships (Figure 6). For bacterial community, modules contained between 4 and 7 phyla. After cucumber planting, connections and relationships became denser and closer. Dazomet-treated soil had the least connections and interrelationships compared to other treatments. RB41 was not the dominant group in soil treated with dazomet; however, in soil treated with kasugamycin, it was one of the dominant groups and has close and complex interaction with other dominant groups.

Fungal networks showed more connected and closer relationships in fenaminosulf-treated soil than other treatments (Figure 7). In addition, there were more phyla in modules of the non-treated soil than the other treatments. In particular, only three phyla of fungi were detected under fenaminosulf treatment, including Ascomycota, Basidiomycota, and Olpidiomycota. Both positive and negative correlations were observed in modules of the non-treated soil, whereas fewer negative correlations were found in modules of disinfectant-treated soil. Furthermore, no negative correlation was found in the module of dazomet-treated soil (Figure 7). Disinfectants strengthened the interaction of potential biocontrol agents, such as *Penicillium* and *Trichoderma* spp., and plant pathogens (Al-Jabry et al., 2019; Huang et al., 2020). Fenaminosulf and dazomet application promoted the interaction of *Penicillium* and *Fusarium*, and kasugamycin and dazomet activated the interaction of *Trichoderma* and *Fusarium*. *Mortierella* was the highest relative abundance in the soil except those treated with fenaminosulf. Its relative abundance firstly decreased and then increased after planting cucumber. The relative abundance of *Fusarium* increased gradually depending on cucumber growth.

## Discussion

The purpose of soil treatments with various disinfectants is primarily to control soilborne plant diseases. We have confirmed that fungal species that are previously reported as pathogens such as *Verticillium dahliae*, *Fusarium oxysporum*, and *Fusarium solani* were suppressed by fenaminosulf and dazomet (Supplementary Figure 1; Emmanouil et al., 2016; Zhao et al., 2017). Meanwhile, some beneficial microorganisms, including pathogen antagonists, increased under soil treatments, as shown by increased relative abundance. Similar results have been observed in apple orchards (Nicola et al., 2017). The increased population of beneficial microorganisms might contribute to the suppression of fungal diseases (Huang et al., 2015). In this study, there were shifts of relative abundance in taxa in disinfectant-treated soil. Kasugamycin and fenaminosulf did not significantly affect soil microbial richness, community structure, and diversity, but dazomet did, as shown by others (Nicola et al., 2017; Fang et al., 2020). It was not surprising that fenaminosulf did not affect bacterial community structure because it is a fungus-specific fungicide and only effective on fungi. Likewise, kasugamycin only knocked down some pathogenic bacterial populations, and many bacteria in soil were resistant to kasugamycin (Gayle and George, 2011; Nguyen et al., 2018). In contrast, dazomet is a non-selective chemical and affects most soil bacteria and fungi (Nicola et al., 2017).

Soil microbial community can be measured as an indicator for evaluating the effectiveness of soil restoration from disturbance and stress (Kibblewhite et al., 2008). In this study, relative abundance of microbial communities and co-occurrence networks were significantly changed by all test disinfectants. Microbial taxa, average degree, and edges showed obvious differences within these networks. Disinfectant application has a certain impact on interspecific relationship of microbial communities. After soil treatment, cucumber cultivation brought up a few new phyla of fungi. The changes of co-occurrence networks imply the shifts of microbial associations at different sampling times under disinfectant application. This suggested that both disinfectant application and plant cultivation influenced soil microbial interspecific relationships.

Disinfectants can change microbial communities, but they also can be degraded by certain microorganisms (Lei et al., 2012). For example, kasugamycin is easily degraded, and dazomet in soil is quickly converted to methyl isothiocyanate (Lei et al., 2012; López-Fernández et al., 2016). This explained why kasugamycin and dazomet were undetectable in soil 14 days after disinfectant application. The recovery of soil microbes is closely linked to the persistence of residual fumigants in the soil (Fang et al., 2020). For example, chloropicrin has a half-life of approximately 0.2–4.5 days, but microbial activity did not recover until day 59 in chloropicrin-fumigated soil (Gan et al., 2000; Fang et al., 2020). Therefore, rapid degradation of disinfectants in soil contributed to the recovery of the microbial community.

Many soil microorganisms are active in nutrient cycling and plant nutrient availability by producing various enzymes (Kiss and Simihăian, 2002). As such, enzymatic activity of these microbes can be used as an indicator of environmental stability and soil health (Kibblewhite et al., 2008). Pesticides reduce soil microorganisms, which can be indicated by reduced enzymatic activities (Li et al., 2017). In this study, dazomet significantly inhibits the activity of UE and SC of soil microbiota, which is in agreement with others (Bremner, 1995; Kiss and Simihăian, 2002; Xu et al., 2018). Kasugamycin has been reported to inhibit UE activity in the beginning but enhance the activity at a later stage (He et al., 2010). We observed that UE activity was decreased by kasugamycin application, but the reduced population significantly increased in the presence of cucumber, indicating a quick recovery by plant cultivation.

Chemical properties of soil are closely related to the changes of microorganisms (Congcong et al., 2015). Soil TN influences C sequestration in terrestrial ecosystems (Wang et al., 2015). Addition of pesticides to soil can result in unwanted increases in C and N supply to surviving microbes due to a pulse in microbial necromass (Mohammad et al., 2021). We have found that TN and TC contents significantly increased in dazomet-treated soils at the later stage after cucumber planting, as documented by others (Bonanomi et al., 2008). The increase of TN might be related to relative abundance changes of N-cycling bacteria. Nitrospira significantly decreased in dazomet-treated soil but increased when cucumber was cultivated, which is negatively correlated to nitrite concentrations (Daims et al., 2015). Relative abundances of the N_2_-fixing bacteria *Bradyrhizobium* and denitrification bacteria such as *Streptomyces*, *Sphingomonas*, and *Pseudomonas* spp. are higher in dazomet-treated soil than those in other treatments (Fang et al., 2018). The shifts of microbial population associated with N cycles resulted in the change of N content in soil.

The enzymatic and chemical measurements may not be aligned to the variations of microbial communities. Microbial communities contain different microbes that have various biological functions. Disinfectant application and cucumber cultivation only influence some functional microorganisms, but not all. Another possibility is that the proportion of different functional microorganisms in the microbial population is variational. Variation of functional microorganisms in the treatments may not be large enough to affect the change of the total microbial population (Cao et al., 2021).

Plants promote soil microbial restoration by root exudation (Tao et al., 2020; Miaoping et al., 2021). Root exudates play a vital role in regulating colonization of rhizosphere microbes and simultaneously activating indigenous microbes associated with plant growth (Yunpeng et al., 2017; Kaur et al., 2021). Root exudates are an important source of nutrients to soil microorganisms. Some compounds in the root exudates possibly function as signals to attract plant growth-promoting rhizobacteria and promote their colonization that in turn suppresses soilborne diseases (Yunpeng et al., 2017). These colonized cucumber roots reduce raffinose secretion to inhibit root colonization of *F. oxysporum* (Yunpeng et al., 2017). Furthermore, the *Fusarium*-susceptible cucumber tends to recruit more beneficial microbes such as *Comamonadaceae*, *Pseudomonas*, and *Stenotrophomonas* through secreting more organic acids, such as citric acid, pyruvate acid, succinic acid, and fumarate, compared to *Fusarium*-resistant cucumber (Yao and Wu, 2010; Tao et al., 2020). Our study indicated that cucumber cultivation promoted the relative abundance of *Streptomyces* and *Trichoderma* spp., which are common biocontrol agents. The biomass of root exudates and the decomposition of senesced roots contribute to the growth and activity of microbes (Miaoping et al., 2021). Therefore, plants will actively cooperate with microorganisms to control pathogens and protect the healthy growth of plants.

Disinfectants caused significant changes of chemical properties, enzymatic activities, and microbial communities in soil. These changes, however, tended to be evened after planting cucumber, suggesting that soil restoration might link to the presence of crops. The enzymatic activity recovered quickly in kasugamycin- and fenaminosulf-treated soils but delayed in dazomet-treated soil. That was because dazomet had a significant reduction of all microbes, resulting in a longer time for the community to recover, whereas kasugamycin and fenaminosulf partially affected the communities, on either bacteria or fungi only, resulting in a relatively faster recovery of soil microbial communities.

## Conclusion

Disinfectants had various impacts on fungal and bacterial communities in soil, depending on their spectrum of effectiveness. Kasugamycin and fenaminosulf suppressed certain types of microbes, but dazomet affected both fungi and bacteria. The influenced organisms included plant pathogens, mutualists, and beneficial organisms. Soil microbial communities were significantly affected in relative abundance and diversity, but not compositional structures of the predominant bacterial and fungal groups. Regardless, plant cultivation might contribute to soil restoration under the stress of disinfectants.

## Data Availability Statement

The datasets presented in this study can be found in online repositories. The names of the repository/repositories and accession number(s) can be found below: https://www.ncbi.nlm.nih.gov/, PRJNA392356.

## Author Contributions

YW was responsible for experimental design and manuscript preparation. JH was responsible for writing guidance. YJ and PH were responsible for experiment performance. HP and JL were responsible for experimental data processing and analysis. All authors contributed to the article and approved the submitted version.

## Conflict of Interest

The authors declare that the research was conducted in the absence of any commercial or financial relationships that could be construed as a potential conflict of interest.

## Publisher’s Note

All claims expressed in this article are solely those of the authors and do not necessarily represent those of their affiliated organizations, or those of the publisher, the editors and the reviewers. Any product that may be evaluated in this article, or claim that may be made by its manufacturer, is not guaranteed or endorsed by the publisher.
