# Supplementary material for: Impact of Soil Disinfestation on Fungal and Bacterial Communities in Soil With Cucumber Cultivation
Source: Front Microbiol. 2021 Aug 19;12:685111. doi: 10.3389/fmicb.2021.685111 (PMC8417054; doi:10.3389/fmicb.2021.685111)
Supplement: Supplementary file 1 [file Data_Sheet_1.docx]

Table S1 Accuracy of extraction methods for disinfectants in soil

| Disinfectants | Regression equation | *R^2^* | Fortified level  (mg/kg) | Detection limit  （mg/L） | Recovery rate  (%) | RSD *  (%) |
| --- | --- | --- | --- | --- | --- | --- |
| Fenaminosulf | y = 31.047x + 2.932 | 0.9997 | 1 | 0.017 | 92.0 | 1.9 |
|  |  |  | 10 |  | 76.5 | 4.4 |
| Kasugamycin | y = 17.525x - 27.959 | 0.9975 | 10 | 0.085 | 69.4 | 4.1 |
|  |  |  | 50 |  | 72.7 | 3.3 |
| Dazomet | y = 85.576x + 0.9386**^＃^** | 0.9998 | 2 | 0.021 | 95.5 | 10.2 |
|  |  |  | 10 |  | 80.7 | 5.7 |

* Relative standard deviation, **^＃^**x represents residue concentration of disinfectants and y represents peak area measured by high performance liquid chromatography (HPLC).

Table S2 Relative abundance of bacterial and fungal phyla, 2018

|  | Phyla | The relative abundance (%) | | | | | | | | | | | |  |
| --- | --- | --- | --- | --- | --- | --- | --- | --- | --- | --- | --- | --- | --- | --- |
|  |  | A1^x^ | B1 | C1 | D1 | A2 | B2 | C2 | D2 | A3 | B3 | C3 | D3 | |
| Bacteria | Chloroflexi | 14.11ab | 16.32a | 13.41ab | 13.05ab | 8.24b | 8.72b | 10.34b | 12.36ab | 12.00ab | 11.18ab | 10.36b | 12.74ab | |
|  | Actinobacteria | 19.17a | 17.72a | 18.13a | 16.83a | 7.27b | 10.36b | 7.68b | 13.26ab | 9.47b | 7.20b | 8.10b | 16.30a | |
|  | Acidobacteria | 18.81bc | 18.86bc | 17.58bc | 13.40c | 21.62b | 25.17ab | 28.00ab | 11.62c | 22.13b | 30.69a | 24.60ab | 11.14c | |
|  | Proteobacteria | 32.65ab | 29.54b | 34.48ab | 38.88a | 38.38a | 32.44ab | 32.37ab | 36.45a | 32.56ab | 30.05b | 33.81ab | 31.34b | |
|  | Total | 84.74a | 82.43a | 83.61a | 82.16a | 75.51ab | 76.69a | 78.39a | 73.69b | 76.17ab | 79.12a | 76.87ab | 71.52b | |
| Fungi | Mortierellomycota | 29.87b | 26.83b | 42.97a | 38.63a | 18.38c | 11.20d | 14.02cd | 11.75cd | 6.81d | 7.28d | 7.80d | 15.78c | |
|  | Ascomycota | 58.62bc | 66.91b | 47.58c | 50.62c | 63.20b | 79.73a | 66.82b | 67.00b | 74.05a | 78.25a | 72.60ab | 66.11b | |
|  | Total | 88.49a | 93.74a | 90.55a | 89.25a | 81.58b | 90.93a | 80.84b | 78.74b | 80.85b | 85.54ab | 80.41b | 81.89ab | |

^x^Treatments:A, non-treated; B, fenaminosulf; C, kasugamycin; D, dazomet. Numbers followingtreatment letters represent sampling dates: 1 =14 d after soil treatment, 2 = 55d after soil treatment, and 3 = 110d after soil treatment.

TableS3 Correlation between soil properties and bacterial and fungal community structures

|  | Bacteria | | Fungi | |
| --- | --- | --- | --- | --- |
|  | r | *P* | r | *P* |
| TN | 0.0329 | 0.256 | 0.1190 * | 0.013 |
| TC | 0.0366 | 0.239 | 0.0976 | 0.052 |
| C/N | 0.0238 | 0.322 | -0.0520 | 0.746 |
| pH | 0.1970 * | 0.013 | 0.0816 | 0.157 |

The correlation coefficient (r) and significance (*P*) were determined by Mantel tests based on 10,000 permutations between community structure (Bray-Curtis dissimilarity) and environmental variables (Standardized Euclidean distance). * indicates significance of r (*P*< 0.05).

Table S4 Network properties of soil bacterial and fungal communities

| Classified | Figure | Average degree | Edge density | Clustering coefficient | Nodes | Edges |
| --- | --- | --- | --- | --- | --- | --- |
| Bacteria | a | 2.8235 | 0.1794 | 0.4918 | 17 | 24 |
|  | b | 3.7777 | 0.2222 | 0.7073 | 17 | 24 |
|  | c | 4.4000 | 0.3143 | 0.6257 | 15 | 34 |
|  | d | 3.0769 | 0.2564 | 0.4705 | 13 | 21 |
|  | e | 2.4285 | 0.1868 | 0.4736 | 14 | 17 |
|  | f | 3.2500 | 0.2167 | 0.5384 | 16 | 28 |
|  | g | 6.2352 | 0.3897 | 0.7124 | 17 | 51 |
| Fungi | a | 3.1578 | 0.1754 | 0.5684 | 19 | 32 |
|  | b | 6.0000 | 0.3750 | 0.7435 | 17 | 46 |
|  | c | 3.3750 | 0.2250 | 0.5806 | 16 | 29 |
|  | d | 2.6667 | 0.1569 | 0.4918 | 18 | 25 |
|  | e | 1.3846 | 0.1153 | 0.0234 | 13 | 9 |
|  | f | 3.6000 | 0.4000 | 0.5000 | 10 | 19 |
|  | g | 2.1538 | 0.1794 | 0.5714 | 13 | 14 |
