# Supplementary figures and images for: Impact of Soil Disinfestation on Fungal and Bacterial Communities in Soil With Cucumber Cultivation
Source: Front Microbiol. 2021 Aug 19;12:685111. doi: 10.3389/fmicb.2021.685111 (PMC8417054; doi:10.3389/fmicb.2021.685111)

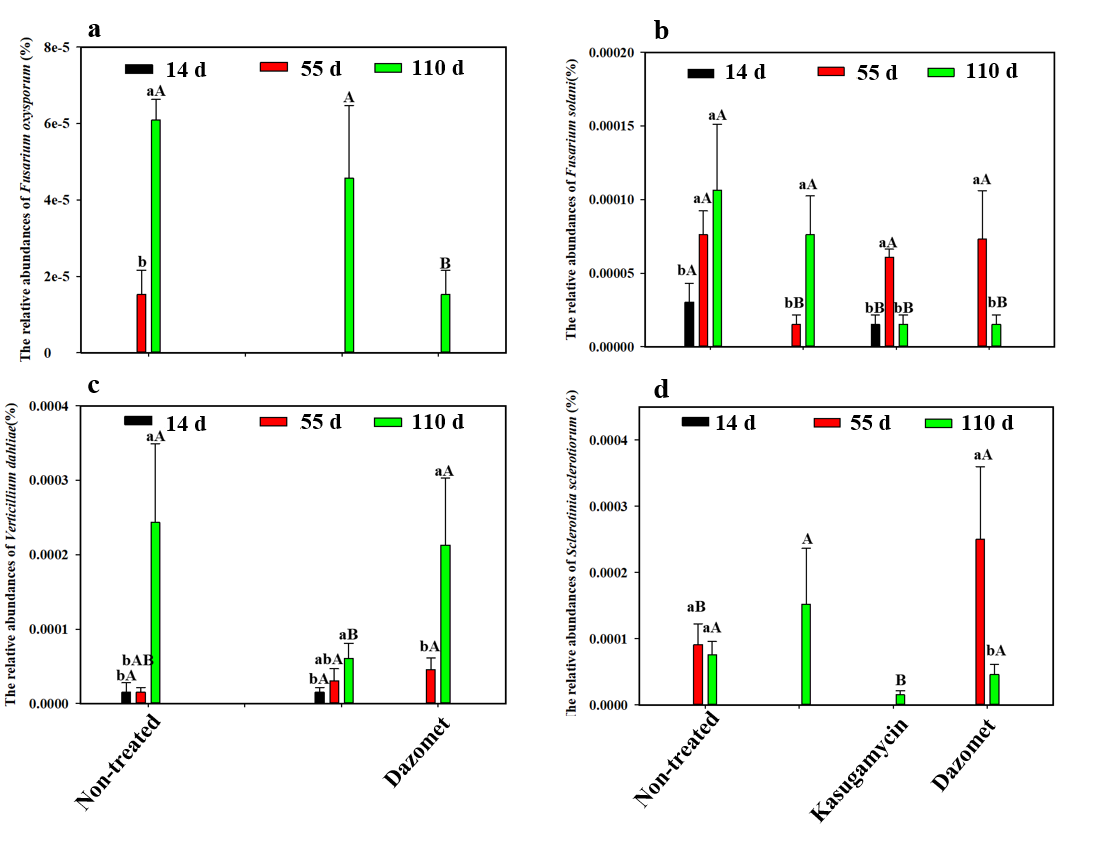

Supplement: Supplementary file 2 [file Image_1.TIF]
